# Editorial: Advances and innovative discoveries in oral potentially malignant disorders

**DOI:** 10.3389/froh.2025.1688292

**Published:** 2025-09-08

**Authors:** Xin-Jia Cai, Saman Warnakulasuriya

**Affiliations:** 1 Central Laboratory, Peking University School and Hospital of Stomatology & National Center for Stomatology & National Clinical Research Center for Oral Diseases & National Engineering Research Center of Oral Biomaterials and Digital Medical Devices & Beijing Key Laboratory of Digital Stomatology & Research Center of Engineering and Technology for Computerized Dentistry Ministry of Health & NMPA Key Laboratory for Dental Materials, Beijing, China; 2 King’s College London, London, United Kingdom

**Keywords:** oral potentially malignant disorders, discoveries, advances, update, editorial

## Introduction

Oral potentially malignant disorders (OPMD) comprise a group of heterogenous disorders that increase the risk of developing oral squamous cell carcinoma (OSCC) (1, 2). It is imperative to identify the clinico-pathologic and molecular factors that increase the risk of malignant transformation, to gain insight into the involved mechanistic pathways and to implement risk stratification in order to facilitate the implementation of early intervention strategies for the management of OPMD (3, 4). However, our understanding of the natural history of OPMD has not been fully elucidated, and the precise strategies based on the integration of various predictive markers have yet to be validated by longitudinal follow up studies and clinical trials (5–8). It is therefore essential to gain a deeper understanding of the complex factors involved in the malignant transformation of OPMD, in order to develop clinically effective preventive and intervention strategies. This special issue in Frontiers compiles six publications that collectively enhance our comprehension of OPMD, spanning biomarkers of malignant transformation, artificial intelligence (AI)-assisted diagnosis, treatment efficacy, and systemic disease associations.

Three articles in this issue delve into molecular and immune microenvironment markers predictive of OPMD progression. López-Pintor et al. investigated salivary lactate dehydrogenase (sLDH) levels in OSCC, OPMDs, and controls. While sLDH levels in saliva were elevated in OSCC patients with larger tumors, no significant differences were observed between OSCC, OPMDs and controls, nor was sLDH correlated with OPMD subtypes or dysplasia severity, suggesting sLDH cannot serve as a standalone biomarker. Complementing this, Cívico-Ortega et al. conducted a meta-analysis revealing EGFR upregulation as a predictor of OPMD transformation (relative risk = 2.17, 95%CI = 1.73–2.73), with protein overexpression, gene amplification, and nuclear staining all showing statistically significant associations. The equivalence between immunohistochemistry and FISH techniques underscores EGFR's clinical utility as a cost-effective risk biomarker. Further mechanistic insights were provided by Sutera et al., whose systematic review delineated macrophage polarization shifts during OPMD-OSCC progression. An M1-dominant pro-inflammatory profile in OPMDs transitions to M2 pro-tumorigenic polarization in severe dysplasia, were investigated with emerging markers like STAT1 and PD-L1 highlighting immunomodulatory pathways. However, methodological heterogeneity and a paucity of longitudinal studies underscore the need for future studies on the expression of specific markers potentially linked to immunomodulatory pathways to unravel macrophage-specific roles in the microenvironment of oral epithelial dysplasia.

Transitioning from biomarkers to diagnostic innovation, Mirfendereski et al. evaluated AI's potential in OPMD/OSCC detection through clinical image analysis. While computer vision shows promise in binary classification and risk stratification, AI-based image analysis for OSCC/OPMD diagnosis and determining prognosis remains in the nascent stage of technical feasibility (9, 10). Further methodological advancements and rigorous enhancements in study design are required to establish generalizability and clinical applicability. Beyond clinical applicability, implementation workflows and actual patient benefits must be carefully considered. Prospective validation and clinician-technological collaboration are critical to bridge the gap between technical feasibility and clinical implementation.

Therapeutic surgical advancements for treating oral leukoplakia are represented by de Pauli Paglioni et al.’s randomized trial comparing diode laser and scalpel excision. Both modalities achieved comparable pain outcomes, though tongue lesions universally elicited greater discomfort. While laser offered superior early wound healing (7-day follow-up), outcomes converged by three months, affirming both techniques as viable options. The study highlights the need for personalized approaches accounting for OPMDs location and patient's age.

Finally, Tenore et al. explored the intersection of diabetes mellitus (DM) and oral lichen planus (OLP). Their retrospective analysis revealed DM patients present with more symptomatic, atrophic lesions and require intensified management, frequent follow-ups and non-steroidal therapies, likely compounded higher rates of cardiovascular and metabolic comorbidities. These findings advocate for interdisciplinary care models integrating endocrinologists to address OLP's systemic drivers.

Collectively, this issue underscores three paradigm shifts in OPMD management: (a) molecular profiling is refining risk prediction but requires standardization; (b) AI and multimodal diagnostics are redefining early detection, pending rigorous clinical validation; and (c) therapeutic personalization, whether selecting evidence-based protocols or addressing systemic comorbidities, is emerging as a cornerstone of care. Future research must prioritize longitudinal designs, biomarker mechanistic studies, and technology implementation frameworks to translate these advances into improved patient outcomes to reduce the global burden of oral cancer.
